# Improving healthcare systems and services in the face of population ageing: policy considerations for low- and middle-income countries

**DOI:** 10.11604/pamj.2022.43.190.30562

**Published:** 2022-12-13

**Authors:** Nnamdi Stephen Moeteke

**Affiliations:** 1 Delta State University Teaching Hospital, Oghara, Delta State, Nigeria

**Keywords:** Population ageing, health systems, developing countries

## Abstract

Worldwide, the proportion and absolute numbers of over 60-year-olds in the population is rising: it was 1 billion in 2019 and is expected to get to 1.4 billion in 2030 and 2.1 billion by 2050, with the progression being more pronounced in developing countries. Degenerative and non-communicable diseases are more common with ageing, which means they would become the major disease problems for countries. Long-term care poses financial risks to individuals/families and governments. These warrant urgent policy and structural adjustments of health systems in low- and middle-income countries to cater for the probable change in health needs and make their society more age-sensitive. There is the need for policymakers to begin to change focus from traditional models of healthcare delivery and give more attention to aged care and create separate financing mechanisms/programmes for the elderly, most of whom are already unemployed, to protect them from the effects of cream-skimming by private health insurers. Informed decisions on healthcare purchasing can be made using Economic Evaluation, as well as Programme Budgeting and Marginal Analysis, a presentation of discrete categories of healthcare expenditure (specialties, disease-groups, etc.) and the resources appropriated to each as an aid to reviewing subsequent resource allocation. Reforms in healthcare financing should pay particular attention to the elderly considering that technical efficiency concerns of the private sector may mean that they are discriminated against. There is need for retraining/reorientation of health workers on identification and comprehensive management of NCDs, including palliative care, in a culturally competent way, on the essence of shifting from an acute care model to a chronic care model, and on providing services to people within defined catchment areas.

## Perspectives

### Introduction/problem statement

Worldwide, the proportion/absolute numbers of over 60-year-olds in the population is rising: it was 1 billion in 2019, and is expected to get to 1.4 billion in 2030 and 2.1 billion by 2050, with the rise being more pronounced in developing countries [1]. Population ageing, which arises from a progressive decrease in fertility and the size of younger age groups accompanied by increased longevity, has become a global public health concern because of its effects on healthcare and social policy. Increased survival means that people go through longer periods of ill-health and need for treatment, leading to increased health spending and pressure on health systems. Degenerative and non-communicable diseases are more common with ageing, which means they would become the major health problems for countries. With more people in the elderly bracket and fewer young adults, the latter would find themselves with few or no siblings to support in catering for elderly parents making institutionalised care a necessity [2].

However, the resultant higher expenditures are not necessarily the fallout of ageing per se but the result of embracing new and expensive technologies and the re-organisation of the health system [3]. Long-term care poses financial risks to individuals/families and governments. It has been projected that federal governments will eventually relinquish the financial burden to States and citizens [4]. The elderly often are in rural areas where health services are most basic, and providers are poorly experienced/equipped in geriatric care since ageing is nascent in developing countries [3]. There is also the possibility of failure to provide adequate access to quality care in other aspects of health and for other segments of the population arising from the competing demands for resources from technology-intensive therapies for advanced Non-Communicable Diseases (NCDs) [5]. All these warrant urgent policy and structural adjustments of health systems in low- and middle-income countries to cater for the probable change in health needs and make their society more age-sensitive [1].

**Health needs assessment:** there is the need for policy-makers to begin to change focus from traditional models of healthcare delivery and give more attention to aged care, and create separate financing mechanisms/programmes for the elderly, most of whom are already unemployed, to protect from the effects of cream-skimming by private health insurers [3]. Of priority is the elimination of long waiting lists for specialised ambulatory care, radiological investigations and surgical procedures, and transfer of cases of acute exacerbations to outpatient clinic, while ensuring adequate access to quality general out-patient services and basic surgical procedures by countering the competing demand for resources by high-technology interventions for advanced NCDs [5]. As states assume increased responsibility for financing long-term care in the midst of dwindling federal allocations, they will have to device means to target resources and control costs. The latter can be more easily attained if the economic climate is made more favourable for private insurance for long-term care, employer-sponsored elder care programmes, and residential care alternatives. Individuals and families may increasingly find themselves left with no option but to foot the bills on their own via mechanisms such as long-term care insurance [4].

### Critical analysis of evidence and application of theory

*Health financing* is the sum of mechanisms within the health system by which funds are collected, pooled, and used to purchase care for the intended population [6]. It provides an essential nexus for collaboration between health managers/policy-makers, purchasers, providers, and users of services. The responsibility of the regulatory apparatus of government is, therefore, to create the enabling institutional framework for providers to deliver quality care and improve the system´s responsiveness to the expectations of the populace [7].

Generally, sources of funding include individuals, households, employees, employers (firms and corporate bodies), and donor agencies and organisations. Funds could be collected by government agencies, social security agency, community health insurance funds, private insurance funds, or direct payment to providers [7]. The essence of pooling of funds is to evenly distribute financial risk among contributors to the pool [7]. With the out-of-pocket payment method, there is no pooling of funds and distribution of risk, and therefore those who cannot afford care do not receive it or receive it catastrophically [8]. Consumers aim to maximise utility relative to price, so demand increases with lower price and vice versa; producers aim to minimise cost relative to price, therefore, supply decreases with lower price and vice versa. The economic theory of consumer and producer choice can be applied to health and health care, and provides insights into consumer and producer behaviour, as well as causes of failure, in the market for health insurance. One cause of market failure in private health insurance is 'adverse selection'. It gives firms an incentive to engage in “cherry-picking”, providing insurance only to people of low risk, and leaving the high-risk groups uncovered [9].

The concept of *scarcity* stems from the fact that resources are insufficient to meet the wants of individuals/society [8]. Health care resources are also scarce because, due to a fixed budget, there is a limit to the government's spending on health. Therefore, choices have to be made, in both production and consumption, about what amount and combination of health care to produce, how to produce it, who pays for it, and how it is distributed [9]. *Opportunity cost* is the benefit not gained because of spending resources on the next best alternative [9]. The opportunity cost of providing long-term care for the elderly and people with NCDs would, therefore, be other forms of healthcare and the utility that could have accrued from them. This brings to the fore the issue of allocative efficiency which is using the combination/deployment of resources that provides the maximum benefit to the whole population; and technical efficiency - the use of the least amount of resources to produce these health outcomes [8]. Every health system aims at providing value for money, as far as is humanly possible, for everyone under the system, and should look at everybody, not just an individual or a group of persons. It would have to be ascertained if providing adequate care for the elderly and chronically ill would achieve allocative efficiency. Priority-setting is especially needed when health systems, as with developing countries, are faced with fixed budgets and heavy disease burden worsened by the epidemiological transition, making it pertinent to adopt health financing optimisation strategies.

Informed decisions on healthcare purchasing can be made using *Programme Budgeting* and *Marginal Analysis*, a presentation of discrete categories of healthcare expenditure (specialties, disease-groups, etc.) and the resources appropriated to each as an aid to reviewing subsequent resource allocation [10]. It organises information on spending patterns to serve as an economic framework for more efficient future health care resource allocation by comparing the outputs of health care services under broad categories/programmes bearing in mind health care objectives and priorities, underpinned by opportunity cost and marginal analysis [11]. Economic evaluation, another tool of normative economics, entails a process of identifying competing interventions, assessing the costs and benefits of each, and comparatively analysing costs and benefits. For example, *Cost Effectiveness Analysis* (CEA) involves comparing relative trade-offs of cost and benefit in a bid to achieve production (economic) efficiency [8]. *Cost Utility Analysis* (CUA), an extended form of CEA, uses *Quality Adjusted Life Years* (QALY) rather than natural units to measure benefits. These can be used in ascertaining what alternative gives the most output for a given cost.

### Recommendations and conclusion

Many western countries like the United Kingdom have strategies in place, having crossed the threshold for aged societies (i.e. at least 14% of the population aged 65 or above) over four decades ago [12]. Developing countries should consider the following in readiness for the imminent pan-global demographic and epidemiological transitions.

a) Health policy reforms to cater for the aged have to be built into a broader framework of inter-sectoral action geared towards improvement in quality of life and socioeconomic wellbeing [3].

b) Health promotion strategies can make a huge impact on the health status of the elderly as preventive interventions such as campaigns on healthy diet and control of tobacco use have been demonstrated to be far more cost-effective than a curative approach and “developing healthy lifestyles and environments for all age groups will mean fewer problems for tomorrow´s aged” [3].

c) Reforms in healthcare financing should pay particular attention to the elderly considering that technical efficiency concerns of the private sector may mean that they are discriminated against [3].

d) There should be investments in a district-based primary healthcare system that integrates management of chronic diseases and cost-effective community-based prevention services into general health services alongside a formidable surveillance system, particularly as the major NCDs have common risk factors - smoking, physical inactivity, harmful use of alcohol, and an unhealthy diet - that are modifiable [13].

e) There is need for retraining/reorientation of health workers on identification and comprehensive management of NCDs, including palliative care, in a culturally-competent way, on the essence of shifting from an acute care model to a chronic care model, and on providing services to people within defined catchment areas [13].

f) Of importance is a change from the traditional paternalistic medical approach to patient-centred management against the backdrop of prolonged care and need for behavioural change and family/communal support [13].

g) Subsidisation or free provision of basic medicines for NCDs such as hypertension, diabetes, osteoarthritis, asthma, etc. [5].

h) Health technology assessment should be enhanced to provide a sound basis for the proper selection of new public health programmes and actions, and of new drugs, devices and diagnostic tests [5].
